# Relationship between coronary artery calcification and myocardial ischemia on computed tomography myocardial perfusion in patients with stable chest pain

**DOI:** 10.1007/s12350-019-01869-8

**Published:** 2019-09-16

**Authors:** Mohammed El Mahdiui, Jeff M. Smit, Alexander R. van Rosendael, J. Wouter Jukema, Jeroen J. Bax, Arthur J. H. A. Scholte

**Affiliations:** Department of Cardiology, Heart Lung Center, Leiden University Medical Center, Albinusdreef 2, 2300 RC Leiden, The Netherlands

**Keywords:** CAD, myocardial ischemia and infarction, CT Atherosclerosis

## Abstract

**Background:**

Coronary artery calcium (CAC) score has shown to provide incremental prognostic information when added to the Framingham risk score. Although the relation between CAC and myocardial ischemia has been evaluated, there has been little evaluation of the relationship between CAC score and inducible myocardial ischemia on computed tomography myocardial perfusion (CTP).

**Methods and Results:**

Patients who were referred with stable chest pain from the outpatient clinic and who underwent non-contrast computed tomography scan, coronary computed tomography angiography, and adenosine stress CTP were included in this study. CAC score was subdivided in four groups (1 to 99; 100 to 399, 400 to 999, and ≥ 1000). Inducible myocardial ischemia was considered when reversible perfusion defects were observed in ≥ 1 segment. A total of 131 patients (age 62 ± 9.4 years; 56% male) were included. The median CAC score was 241 (73 to 539). Forty-nine patients (37%) had evidence of inducible myocardial ischemia. The presence of inducible myocardial ischemia increased with increasing CAC score from 22% in the CAC score 1 to 99 subgroup to 35, 47, and 65% in the 100 to 399, 400 to 999, and ≥ 1000 CAC score subgroup, respectively. In multivariable analysis CAC score was the only determinant that significantly predicted the presence of inducible myocardial ischemia on CTP.

**Conclusions:**

In a population of symptomatic patients, the majority of patients with extensive calcification had evidence of inducible myocardial ischemia on CTP. CAC score was the only independent predictor of inducible myocardial ischemia on CTP.

**Electronic supplementary material:**

The online version of this article (10.1007/s12350-019-01869-8) contains supplementary material, which is available to authorized users.

## Introduction

Coronary artery calcium (CAC) score measures calcification in the coronary arterial wall along the whole coronary artery tree and is a good indicator of the extent of coronary artery disease (CAD).^1^,^2^ CAC score has shown excellent prognostic value in asymptomatic patients and has also shown its prognostic value in patients with stable chest pain.^3^–^14^ The degree of CAC correlates well with inducible myocardial ischemia as assessed on single-photon emission computed tomography (SPECT) myocardial perfusion imaging (MPI).^15^–^17^ Computed tomography (CT) myocardial perfusion (CTP) also provides functional information of coronary stenosis.^18^ Previous studies have advocated not to perform coronary computed tomography angiography (CTA) when high CAC score is present but straight away CTP.^19^,^20^ However, no studies have assessed the direct relation of CAC score and inducible myocardial ischemia on CTP.

Therefore, the aim of the current study is to examine the relation between CAC score and inducible myocardial ischemia on CTP in patients with stable chest pain.

## Methods

### Study Population

The study population consisted of patients with stable chest pain who were referred for cardiac CT from the outpatient clinic between March 2013 until June 2018. Patients with presence of calcium on non-contrast CT scan and subsequently underwent coronary CTA and adenosine stress CTP were included in this study. The updated Diamond-Forrester risk model was used to calculate the pre-test likelihood of CAD.^21^ The imaging protocol design at our center has been reported before.^22^,^23^ Patients with ≥ 1 uninterpretable myocardial segments on CTP were excluded from analysis. Patients with a history of myocardial infarction or revascularization were also excluded from analysis. Contraindications for cardiac CT were atrial fibrillation, renal insufficiency, second or third degree atrioventricular block, known allergy to iodine-containing contrast agents, and pregnancy. Clinical data were prospectively entered into the departmental cardiology information system (EPD-Vision©, Leiden University Medical Center, The Netherlands). The Dutch Central Committee on Human-related Research allows the use of anonymous patient data without previous approval of an institutional review board, provided that the data are acquired for routine patient care. All data used for this study were acquired for clinical purposes.

### Cardiac CT Acquisition

Non-contrast CT, coronary CTA, and CTP were acquired on the same day, using a 320-row volumetric scanner (from 2013 until November 2015 Aquilion ONE, Canon Medical Systems, Otawara, Japan and from November 2015 the Aquilion ONE Genesis Edition, Canon Medical Systems, Otawara, Japan).

Patients were instructed not to consume caffeine products 24 hour before examination since CTP with adenosine might be performed. On the day of examination patients were evaluated 1h prior to CT acquisition, by measuring the patient’s heart rate and blood pressure. Metoprolol, 25 mg up until 150 mg, was administrated orally if a patient’s heart rate exceeded 60 beats per minutes (bpm) and no contraindications were present. Additional metoprolol could be administrated intravenously if the heart rate remained above 60 bpm during scout images.

First, a low dose non-contrast enhanced scan was performed to determine the CAC score. Nitro-glycerine (0.4 mg) was sprayed sublingual prior to coronary CTA. The coronary CTA was performed with the following scan parameters: detector collimation of 320 × 0.5 mm, 350 ms gantry rotation time and temporal resolution of 175 ms for the Aquilion ONE and 275 ms gantry rotation time and temporal resolution of 137 ms for the Aquilion ONE Genesis Edition. Peak tube voltage was between 100 and 135 kV and tube current between 140 and 580 mA, depending on body mass index. The contrast agent (Iomeron 400, Bracco, Milan, Italy) was injected in the antecubital vein. First, 50 to 90 mL (depending on patient weight) contrast agent (flow rate 5 to 6 mL/s) was administrated, followed by 20 mL of a 1:1 mixture of contrast and saline and finally 25 mL of saline (flow rate 3 mL/s). Prospective ECG triggering was used to scan 70 to 80% of the RR-interval, in patients with heart rate > 65 pm, 30 to 80% of the RR-interval was covered. Real-time bolus tracking was performed in the descending aorta, and coronary CTA was performed the next beat when the threshold of 300 Hounsfield units (HU) was reached.

Stress CTP was performed at least 20 minutes after coronary CTA to achieve adequate myocardial contrast wash-out. After 4 minutes of continuous adenosine infusion (0.14 mg/kg/min) and continuous electrocardiogram and blood pressure monitoring, contrast agent was given. After reaching the target threshold of 300 HU in the descending aorta, CTP images were acquired the next heart beat scanning 80 to 99% of the RR-interval. The tube settings, injection protocol, and contrast agent were similar to the coronary CTA acquisition. If side-effects occurred during adenosine infusion, the administration was discontinued which resolved the side-effects rapidly and theophylline or atropine could be administrated if needed. The effective radiation dose was calculated by multiplying the dose-length-product by a conversion coefficient 0.014 mSv/(mGy × cm).^24^

### Image Reconstruction and Analysis

Collected images were transferred to a workstation and analyzed using dedicated post-processing software (Vitrea FX 6.5; Vital Images, Minnetonka, Minnesota). For the assessment of the CAC score, images with a 3 mm slice thickness were reconstructed from the non-contrast CT. To analyze the CAC score, pixels exceeding 130 HU were recognized and encircled in the course of a coronary artery and calculated according to the Agatston method.^1^ CAC score was categorized into 4 subgroups, minimal to mild calcification (CAC score = 1 to 99), moderate calcification (CAC score = 100 to 399), severe calcification (CAC score = 400 to 999) and extensive calcification (CAC score ≥ 1000). For myocardial perfusion analysis, cardiac phases were reconstructed every 2% of the scanned interval. The phase with the best image quality was selected and interpreted with a narrow window width and level setting (W300/L150), according to the standard 17 myocardial segment model.^25^ For per vessel analysis, individual myocardial segments were assigned to the 3 major coronary arteries using also the standard 17 myocardial segment model. After the initial analysis, observers were allowed to adjust the display settings. All images were analyzed and interpreted by two trained observers. For the present analysis CTA images were exclusively used for rest perfusion data and not for stenosis degree and/or plaques analysis. For stress data, CTP images during adenosine infusion were used. CTP images were arranged in the short axis, vertical long axis and horizontal long axis with a slice thickness of 3 mm. Each segment was scored for perfusion defects and if present, other phases were checked to differentiate between real perfusion defects or artifacts.^26^ When perfusion defects were observed in ≥ 1 segment, the CTP was considered abnormal. Summed difference score (SDS) was calculated by subtracting the summed rest score (SRS) from the summed stress score (SSS). To calculate the SRS and the SSS, all abnormal segments were added from the rest data and stress data, respectively. Inducible myocardial ischemia was defined by a SDS ≥ 1.

### Statistical Analysis

Continuous variables are depicted as mean ± standard deviation when normally distributed and median with 25 to 75th percentile (interquartile range (IQR)) when non-normally distributed. Normally distributed variables were analyzed using the independent sample *t* test and non-normally distributed variables using the Mann Whitney *U* test or the Kruskal Wallis. Summed scores are depicted as mean and range. Categorical variables are depicted as percentages and numbers and analyzed using the *χ*^2^ test. Correlation between CAC score as a continuous variable and extent of myocardial ischemia as assessed by SDS was tested with the Spearman correlation coefficient. Univariable and multivariable analysis were performed to evaluate the variables that were significantly associated with myocardial ischemia on CTP. Variables with a *P* value < 0.1 in univariable analysis and age and gender were included in the multivariable analysis. A *P* value < 0.05 was considered statistically significant. All statistical analyses were performed using SPSS version 23.0 (SPSS, Armonk, NY).

## Results

### Clinical Characteristics

A total of 146 patients were identified. One patient was revascularized and was excluded from further analysis. We excluded 14 patients because of 1 or more uninterpretable myocardial segments on CTP, leaving 131 patients (age 62 ± 9.4 years; 56% male) for analysis. Clinical characteristics are shown in Table 1. Prevalence of cardiovascular risk factors was high in the total population. There were no differences between both groups regarding the presence of cardiovascular risk factors, pre-test likelihood, or medication use.

**Table 1:** Clinical characteristics divided according to the presence of inducible myocardial ischemia

	Total population (n = 131)	Myocardial ischemia (n = 49)	No myocardial ischemia (n = 82)	*P* value
Age (years)	62 ± 9.4	63 ± 7.9	61 ± 10.2	0.332
Male, n (%)	73 (56)	27 (55)	46 (56)	0.912
BMI (kg/m^2^)	27 ± 4	26 ± 4	27 ± 5	0.449
Cardiovascular risk factors				
Current smoking, n (%)	18 (14)	9 (18)	9 (11)	0.296
Diabetes, n (%)	32 (24)	11 (22)	21 (26)	0.684
Family history of CVD, n (%)	67 (51)	27 (55)	40 (49)	0.484
Hypercholesterolemia, n (%)	57 (44)	24 (49)	33 (40)	0.329
Hypertension, n (%)	80 (61)	35 (71)	45 (55)	0.060
Updated Diamond-Forrester risk score (%)				
Intermediate (20–80%) pre-test risk, n (%)	84 (64)	34 (69)	50 (61)	0.376
Medication				
Aspirin, n (%)	35 (27)	14 (29)	21 (26)	0.711
Thienopyridine, n (%)	3 (2)	2 (4)	1 (1)	0.556
OAC, n (%)	14 (11)	6 (12)	8 (10)	0.772
β-blocker, n (%)	70 (53)	30 (61)	40 (49)	0.167
Statin, n (%)	61 (47)	24 (49)	37 (45)	0.668
Diuretic, n (%)	26 (20)	10 (20)	16 (20)	0.901
ACE-I/ARB	55 (42)	23 (47)	32 (39)	0.374

Values are shown as n (%) or as mean ± standard deviation

*ACE-I*, angiotensin-converting enzyme inhibitor; *ARB*, angiotensin receptor blocker; *BMI* body mass index; *CABG*, coronary artery bypass grafting; *CVD*, cardiovascular disease; *OAC*, oral anticoagulants; *PCI*, percutaneous coronary intervention

### Coronary Artery Calcium Score

The median CAC score of the study population was 241 (73 to 539). 41 (31%) patients had minimal to mild calcification (CAC score 1 to 99), 43 (33%) had moderate calcification (CAC score 100 to 399), 30 (23%) had severe calcification (CAC score 400 to 999), while 17 (13%) patients had extensive calcifications (CAC score ≥ 1000).

### CT Myocardial Perfusion

A total of 55 (42%) patients had myocardial perfusion abnormalities at stress, 6 (5%) patients had only perfusion defects at rest and 49 (37%) patients had 1 or more segments with reversible defects on CTP, indicating inducible myocardial ischemia. The mean (range) SRS, SSS, and SDS were 0.22 (0 to 12), 2.26 (0 to 17), and 2.04 (0 to 16), respectively. The median effective radiation dose for CTP was 2.8 mSv (IQR 1.8 to 4.4).

### Relation Between CAC Score and Inducible Myocardial Ischemia on CTP

**Figure 1:**
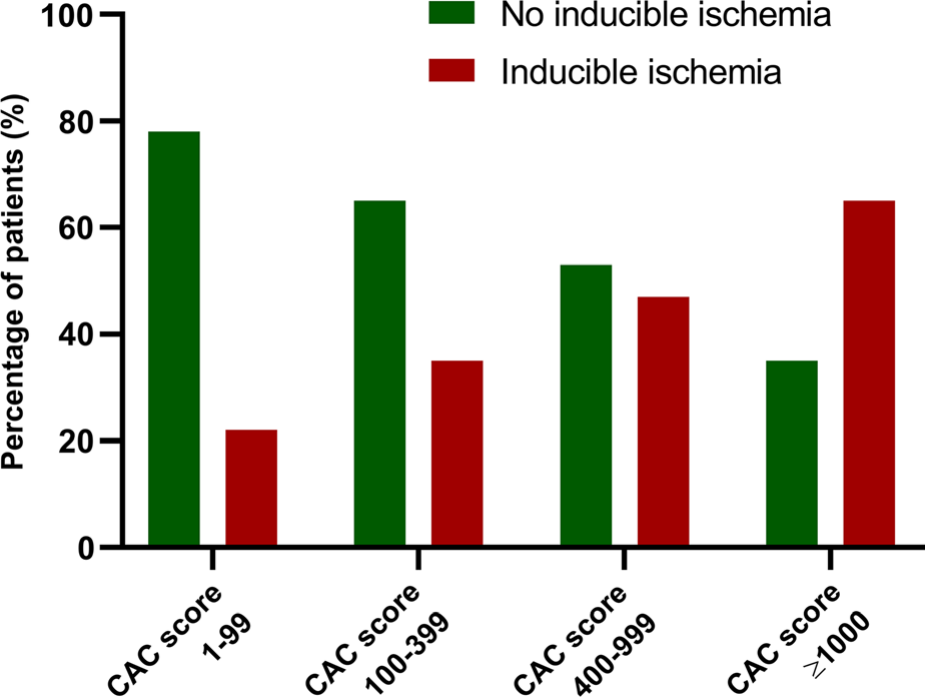
Relationship between percentage of patients with inducible myocardial ischemia and CAC score subgroups. The percentage of subjects with inducible myocardial ischemia on CTP increased with increasing CAC score severity. *CAC score*, coronary artery calcium score

**Figure 2:**
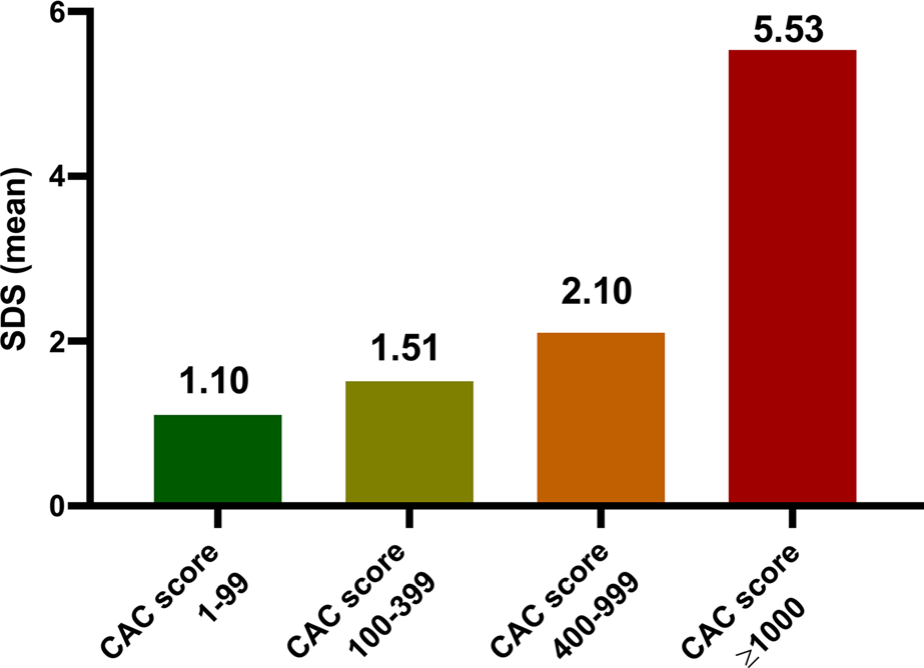
Relationship between extent of inducible myocardial ischemia on CTP and CAC score subgroups. A significant difference was found for the extent of inducible myocardial ischemia between the CAC score subgroups (*P* = 0.002). *CAC score*, coronary artery calcium score; *SDS*, summed difference score

In the subgroups CAC score 1 to 99, CAC score 100 to 399, CAC score 400 to 999, and CAC score ≥ 1000 evidence of inducible myocardial ischemia on CTP was seen in 9 (22%), 15 (35%), 14 (47%), and 11 (65%), respectively. (Figure 1) The relation between CAC score and inducible myocardial ischemia per coronary artery is shown in Table 2. The LAD had higher CAC score independent of the presence of inducible myocardial ischemia or not. The extent of inducible myocardial ischemia (SDS) related to the predefined CAC score subgroups is shown in Figure 2 and the per vessel analysis is shown in Table 3. There was a moderate but significant positive correlation between CAC score and SDS (*r* = 0.368; *P*<0.0001).

**Table 2:** CAC score divided according to the presence of inducible myocardial ischemia

	Total population (n = 131)	Myocardial ischemia (n = 49)	No myocardial ischemia (n = 82)	*P* value
CAC score	241 (73–539)	438 (189–905)	167 (40–421)	0.001*
Per vessel				
CAC score LAD	119 (30–280)	216 (94–415)	84 (21–228)	0.001*
CAC score RCA	24 (0–124)	71 (4–216)	11 (0–84)	0.005*
CAC score LCX	9 (0–101)	34 (1–186)	6.5 (0–73)	0.019*

Values are presented as median (25th–75th percentile)

*CAC score*, coronary artery calcium score; *LAD*, left anterior descending coronary artery; *LCX*, left circumflex coronary artery; *RCA*, right coronary artery

*P < 0.05

**Table 3:** SDS per coronary artery according to the CAC score subgroups

	CAC score 1–99 (n = 41)	CAC score 100–399 (n = 43)	CAC score 400–999 (n = 30)	CAC score ≥ 1000 (n = 17)	*P* value
SDS LAD	0.63 (0–12)	0.67 (0–7)	1.07 (0–6)	3.82 (0–15)	< 0.001*
SDS RCA	0.10 (0–2)	0.42 (0–5)	0.43 (0–2)	1.18 (0–6)	0.013*
SDS LCX	0.37 (0–6)	0.42 (0–4)	0.60 (0–6)	0.53 (0–4)	0.672

Values are presented as mean (minimum–maximum)

*CAC score*, coronary artery calcium score; *LAD*, left anterior descending coronary artery; *LCX*, left circumflex coronary artery; *RCA*, right coronary artery; *SDS*, summed difference score

*P < 0.05

### Uni- and Multivariable Analysis for Inducible Myocardial Ischemia on CTP

The uni- and multivariable analysis for the presence of inducible myocardial ischemia on CTP are shown in Table 4. No cardiovascular risk factors were significantly correlated with inducible myocardial ischemia (Table 3). CAC score was significantly correlated with inducible myocardial ischemia on CTP in univariable analysis (OR 1.001; 95% CI 1.000 to 1.001; *P* = 0.013) and remained significant in the multivariable analysis (OR: 1.001 per 1 Agatston Unit; 95% CI 1.000 to 1.001; *P* = 0.029).

**Table 4:** Uni- and multivariable analysis for inducible myocardial ischemia

	Univariable odds ratio (95% CI)	*P* value	Multivariable Odds ratio (95% CI)	*P* value
Age	1.018 (0.980–1.058)	0.361	1.000 (0.958–1.045)	0.985
Male	0.960 (0.471–1.958)	0.912	0.800 (0.350–1.829)	0.597
BMI	0.968 (0.891–1.052)	0.447		
Current smoking	1.825 (0.671–4.967)	0.239		
Diabetes	0.841 (0.365–1.937)	0.684		
Family history of CVD	1.289 (0.633–2.622)	0.484		
Hypercholesterolemia	1.425 (0.699–2.908)	0.330		
Hypertension	2.056 (0.964–4.383)	0.062	1.626 (0.731–3.618)	0.234
CAC score	1.001 (1.000–1.001)	0.013	1.001 (1.000–1.001)	0.029*

*CAC score*, coronary artery calcium score; *CVD*, cardiovascular disease

*P < 0.05

## Discussion

This study is the first to describe the relationship between CAC score and the presence of inducible myocardial ischemia on CTP. We observed a positive correlation between the burden of CAC and inducible myocardial ischemia on CTP. The frequency of inducible myocardial ischemia on CTP was three times higher for patients with extensive calcification compared to patients with mild calcifications. Moreover, the majority of symptomatic patients referred for CAC scoring with extensive calcifications had inducible myocardial ischemia on CTP.

### CAC Score

Several large retrospective and prospective studies have shown the prognostic value of CAC score measured by coronary CT in asymptomatic patients and its value to improve risk detection over traditional risk factors.^3^–^9^ The prognostic value of CAC score has also been demonstrated in symptomatic patients.^10^–^14^

### CAC Score and CTP

Previous studies applying both CAC score and CTP have primarily focused on the incremental diagnostic value of CTP in the setting of decreased interpretability of coronary CTA in patients with high CAC score.^19^,^20^ Sharma et al. investigated the diagnostic performance of CTP and coronary CTA in 381 patients with intermediate and high risk for CAD and patients with known CAD.^20^ The population was divided in patients with a CAC score 1 to 399 and CAC score ≥ 400. In patients with an CAC score ≥ 400 combined use of coronary CTA and CTP showed superior diagnostic accuracy than coronary CTA or CTP alone, using stenosis ≥ 50% on invasive coronary angiography with corresponding stress perfusion defect on SPECT-MPI as a reference standard. Ladeiras-Lopes et al., reached the same conclusions in a cohort of 95 symptomatic patients with an intermediate pre-test probability of CAD using invasive fractional flow reserve (FFR) as a gold standard.^19^ The direct relationship between CAC score and the presence of inducible myocardial ischemia on CTP has not been investigated before.

### CAC Score and SPECT-MPI

The relationship between CAC and myocardial ischemia on SPECT-MPI has been investigated previously. He et al., showed in a population of 411 predominantly asymptomatic patients that CAC severity was the strongest predictor for the presence of silent myocardial ischemia on SPECT-MPI.^15^ Several studies have shown similar results with increasing rates of myocardial ischemia on SPECT-MPI and PET-MPI with increasing CAC score subgroup.^16^,^17^ A meta-analysis from Bavishi et al., including 20 studies showed a wide range of prevalence of inducible myocardial ischemia on SPECT-MPI or PET-MPI, among the different studies but a consistent increase of inducible myocardial ischemia with increasing CAC score subgroup.^16^ In a large a cohort of 4897 symptomatic patients with low-to-intermediate risk, Engbers et al. showed that CAC score was an independent predictor for abnormal SPECT-MPI.^17^ The frequency of abnormal SPECT-MPI increased with higher CAC score, from 19% in patients with mild calcifications to 50% in patients with extensive calcifications, similar to the results in our study. Interestingly, Engbers et al. also showed that combined evaluation of SPECT-MPI and CAC score provided incremental prognostic information over the individual modalities.^17^ This was also shown by Nappi et al. in a population of 156 patients.^27^ Chang et al. reached the same conclusions in 1126 mostly asymptomatic subjects.^28^ Moreover, in a study by Assante et al., the combined evaluation of CAC score and coronary vascular function as assessed by coronary flow reserve also provided incremental risk stratification for the prediction of adverse cardiac events.^29^

### CAC Score and Other Functional Tests

The relation between CAC score and other functional tests has also been investigated. Ramakrishna et al., investigated the relationship between CAC score and exercise echocardiography in a population of 556 patients.^30^ The correlation between CAC score and exercise wall motion score index (WMSI) was significant, but limited (*r* = 0.17), underscoring the difference between anatomical and functional testing. Patients with both CAC score > 100 and also exercise WMSI > 1 were 4 times more likely to experience a myocardial infarction or die during a follow-up of 5 years compared to patients with a CAC score ≤ 100 and a normal exercise WMSI. Janssen et al., investigated the relation between CAC score and dobutamine cardiovascular magnetic resonance imaging (CMR).^31^ They showed that in a population of 114 symptomatic patients a CAC score ≤ 100 had a negative and positive predictive value of 0.96 and 0.29, respectively, for predicting inducible myocardial ischemia during dobutamine CMR.

CTP has several benefits over SPECT-MPI and other stress testing modalities investigating myocardial ischemia. CTP allows for a fast and simultaneous assessment of anatomical and functional parameters in one session. Furthermore, a recent meta-analysis by Takx et al., showed CTP to accurately rule out hemodynamic significant CAD using invasive FFR as a golden standard, whereas SPECT-MPI and echocardiography were less accurate.^32^

## Limitations

This study has several limitations, inherent to its retrospective and single-center design. The exclusion of patients with uninterpretable CTP imaging might have introduced selection bias. We analyzed the CAC score using the Agatston method and did not incorporate the distribution of the calcifications in the coronary vessel. Although inducible myocardial ischemia has shown prognostic value, in our study we did not look at clinical endpoints.

## Conclusions

In a population of symptomatic patients, the majority of patients with extensive calcification had evidence of inducible myocardial ischemia on CTP. CAC score was an independent predictor of inducible myocardial ischemia on CTP.

## New Knowledge Gained

To the best of our knowledge this is the first study to investigate the direct relationship between CAC score and inducible myocardial ischemia on CTP. Our results suggest that the majority of stable chest pain patients with extensive CAC score have evidence of inducible ischemia on CTP.

## Electronic supplementary material

Below is the link to the electronic supplementary material.

Supplementary material 1 (PPTX 380 kb)

## Abbreviations

CAC: Coronary artery calcium
CAD: Coronary artery disease
CT: Computed tomography
CTA: Computed tomography angiography
CTP: Computed tomography myocardial perfusion
MPI: Myocardial perfusion imaging
SDS: Summed difference score
SPECT: Single-photon emission computed tomography
SRS: Summed rest score
SSS: Summed stress score
