# Supplementary material for: Relationship between coronary artery calcification and myocardial ischemia on computed tomography myocardial perfusion in patients with stable chest pain
Source: J Nucl Cardiol. 2019 Sep 16;28(4):1707–14. doi: 10.1007/s12350-019-01869-8 (PMC8421270; doi:10.1007/s12350-019-01869-8)
Supplement: Supplementary file 1 — Supplementary material 1 (PPTX 380 kb) [file 12350_2019_1869_MOESM1_ESM.pptx]

## Slide 1
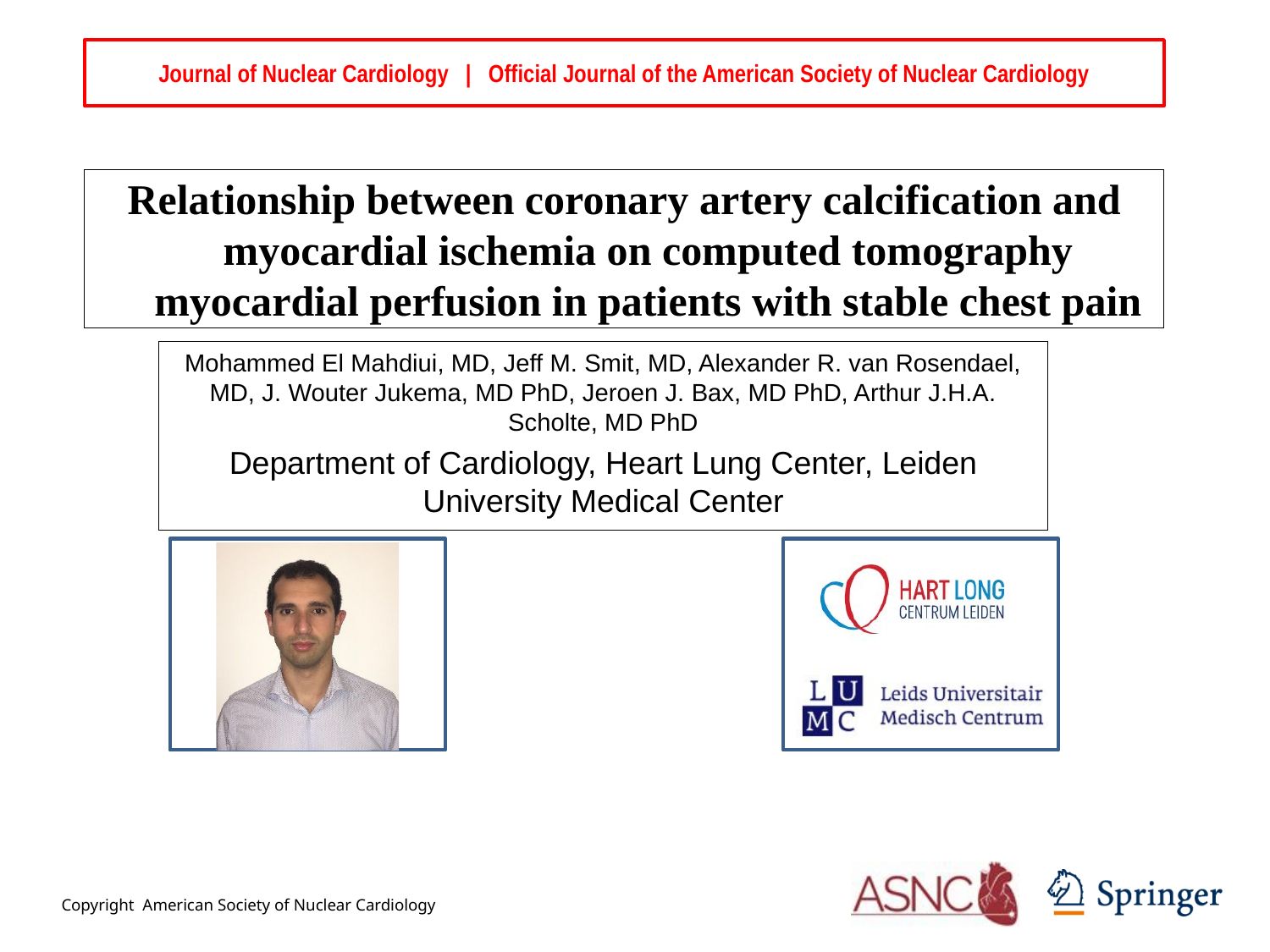

Journal of Nuclear Cardiology | Official Journal of the American Society of Nuclear Cardiology
# Relationship between coronary artery calcification and myocardial ischemia on computed tomography myocardial perfusion in patients with stable chest pain
Mohammed El Mahdiui, MD, Jeff M. Smit, MD, Alexander R. van Rosendael, MD, J. Wouter Jukema, MD PhD, Jeroen J. Bax, MD PhD, Arthur J.H.A. Scholte, MD PhD
Department of Cardiology, Heart Lung Center, Leiden University Medical Center
Head shot of author
required
Copyright American Society of Nuclear Cardiology

## Slide 2
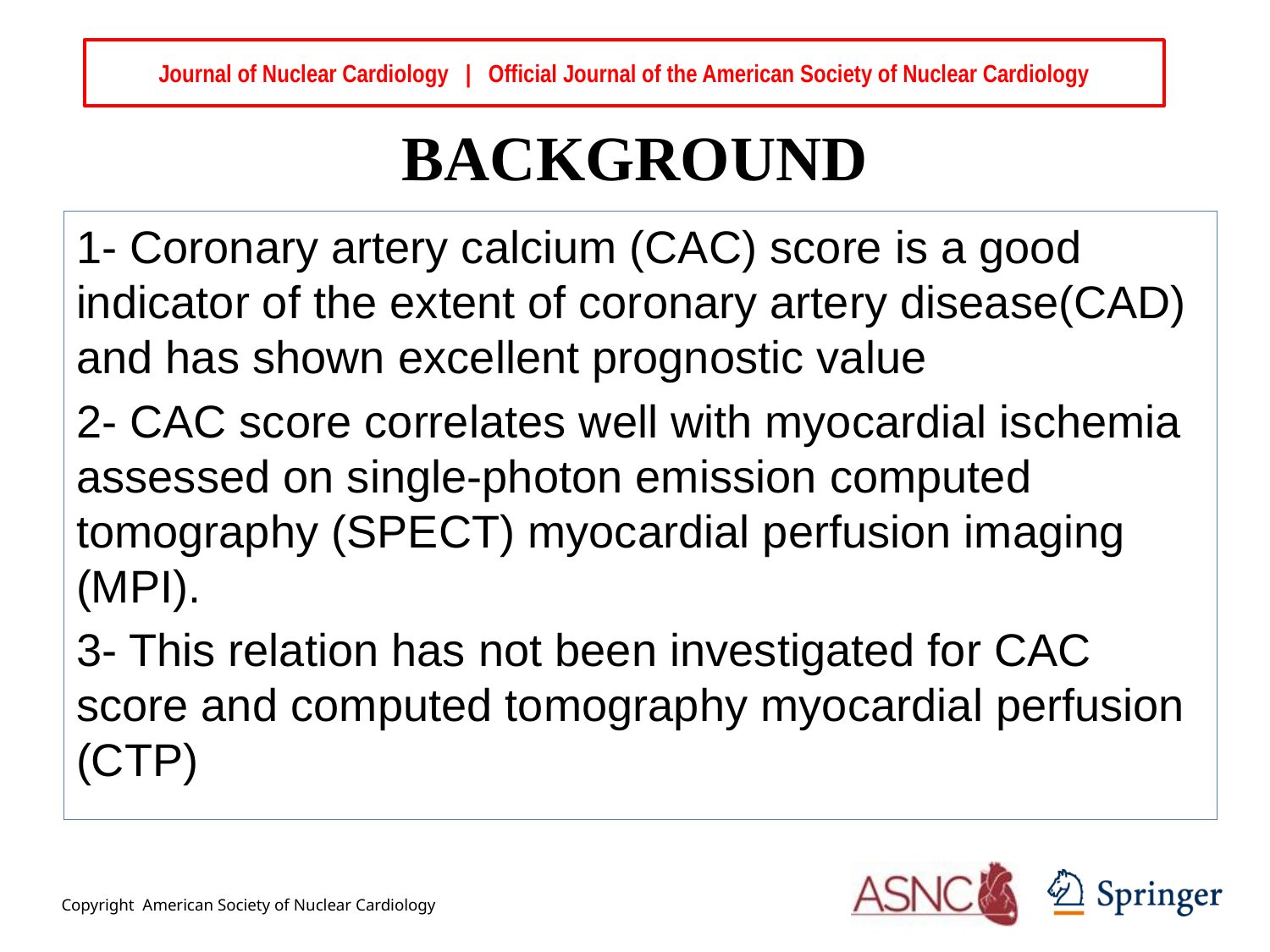

Journal of Nuclear Cardiology | Official Journal of the American Society of Nuclear Cardiology
# BACKGROUND
1- Coronary artery calcium (CAC) score is a good indicator of the extent of coronary artery disease(CAD) and has shown excellent prognostic value
2- CAC score correlates well with myocardial ischemia assessed on single-photon emission computed tomography (SPECT) myocardial perfusion imaging (MPI).
3- This relation has not been investigated for CAC score and computed tomography myocardial perfusion (CTP)
Copyright American Society of Nuclear Cardiology

## Slide 3
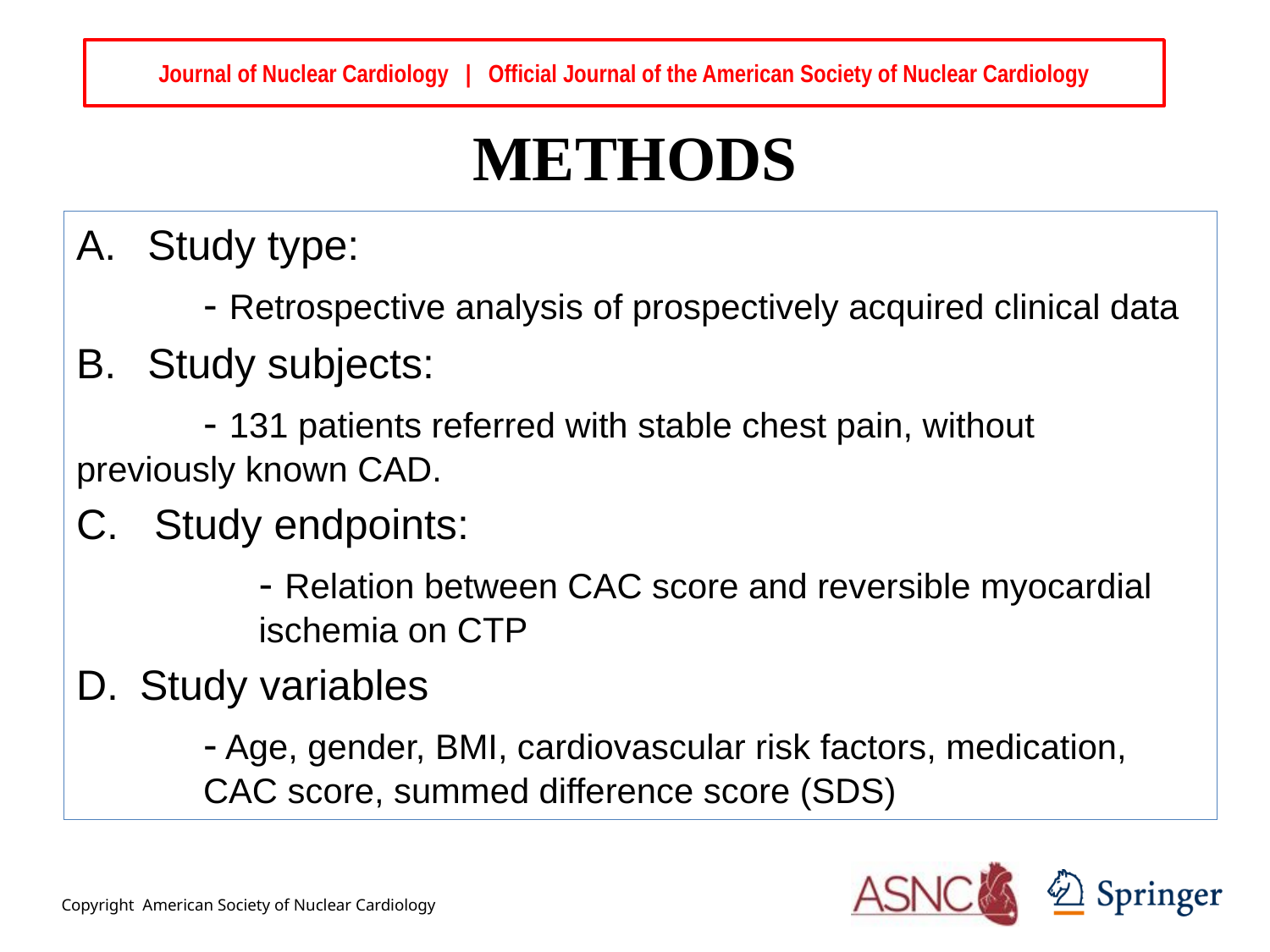

Journal of Nuclear Cardiology | Official Journal of the American Society of Nuclear Cardiology
# METHODS
Study type:
	- Retrospective analysis of prospectively acquired clinical data
Study subjects:
	- 131 patients referred with stable chest pain, without 	previously known CAD.
C. Study endpoints:
	- Relation between CAC score and reversible myocardial 	ischemia on CTP
Study variables
	- Age, gender, BMI, cardiovascular risk factors, medication, 	CAC score, summed difference score (SDS)
Copyright American Society of Nuclear Cardiology

## Slide 4
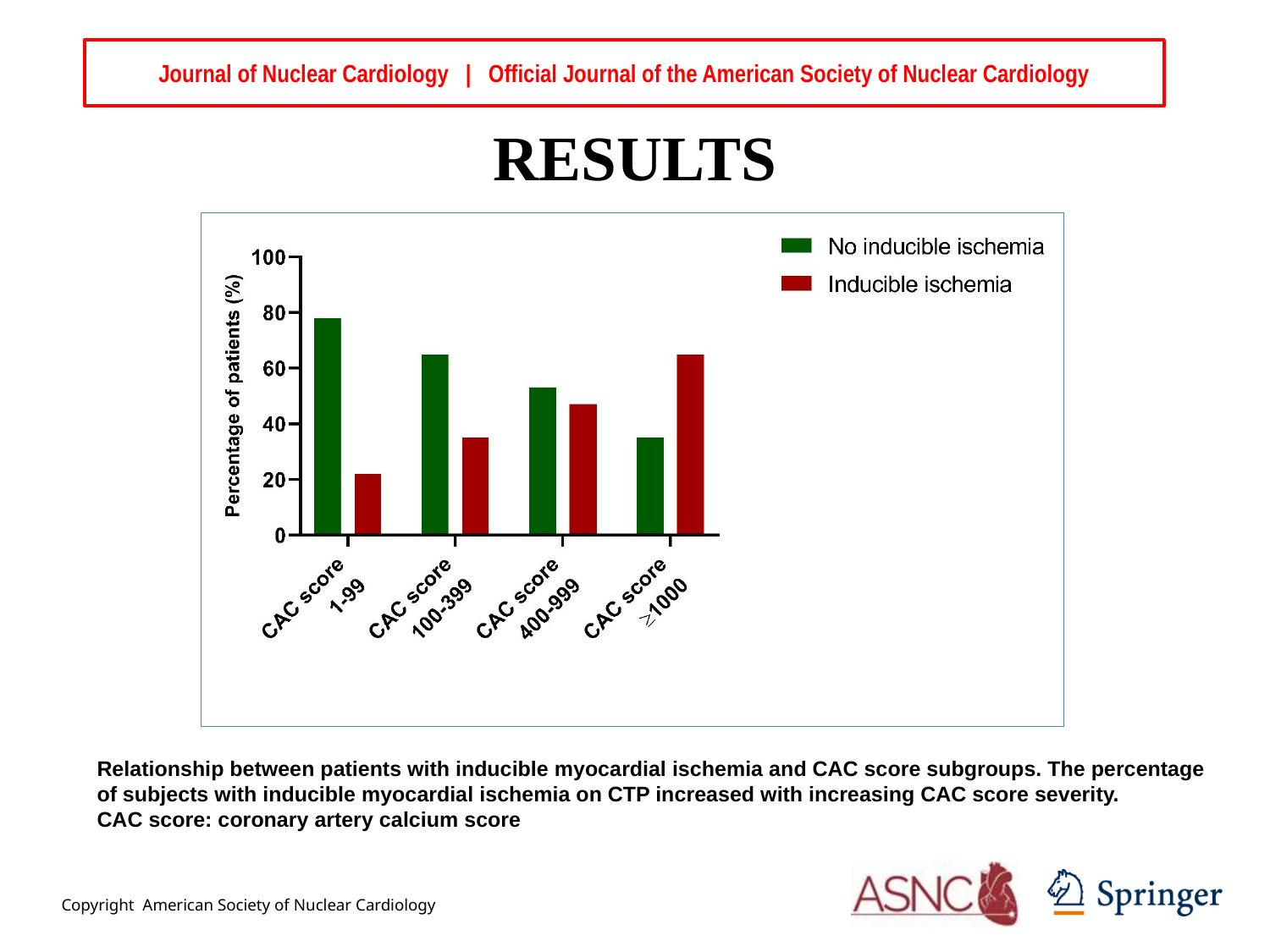

Journal of Nuclear Cardiology | Official Journal of the American Society of Nuclear Cardiology
# RESULTS
Relationship between patients with inducible myocardial ischemia and CAC score subgroups. The percentage of subjects with inducible myocardial ischemia on CTP increased with increasing CAC score severity.
CAC score: coronary artery calcium score
Copyright American Society of Nuclear Cardiology

## Slide 5
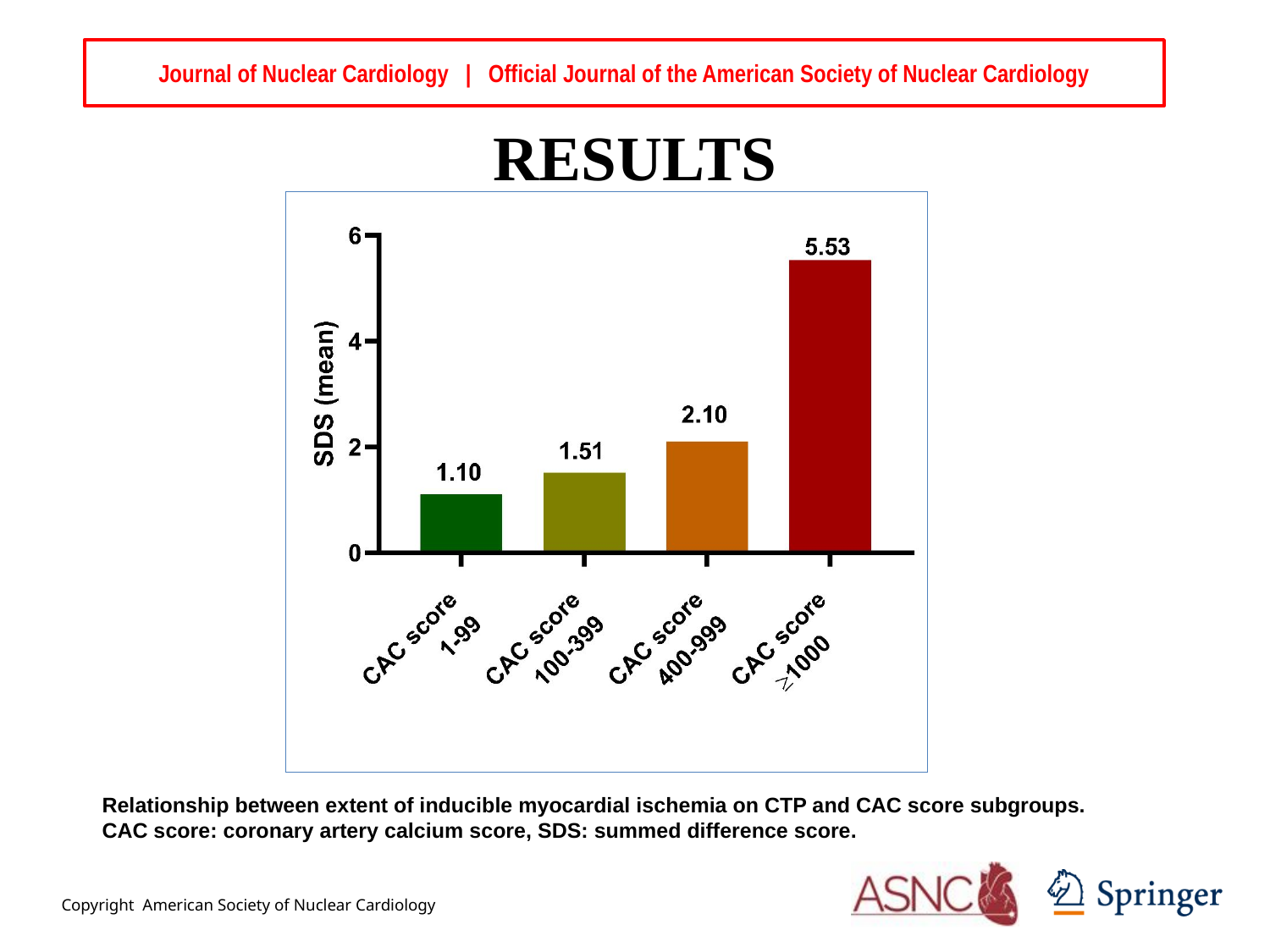

Journal of Nuclear Cardiology | Official Journal of the American Society of Nuclear Cardiology
# RESULTS
Relationship between extent of inducible myocardial ischemia on CTP and CAC score subgroups.
CAC score: coronary artery calcium score, SDS: summed difference score.
Copyright American Society of Nuclear Cardiology

## Slide 6
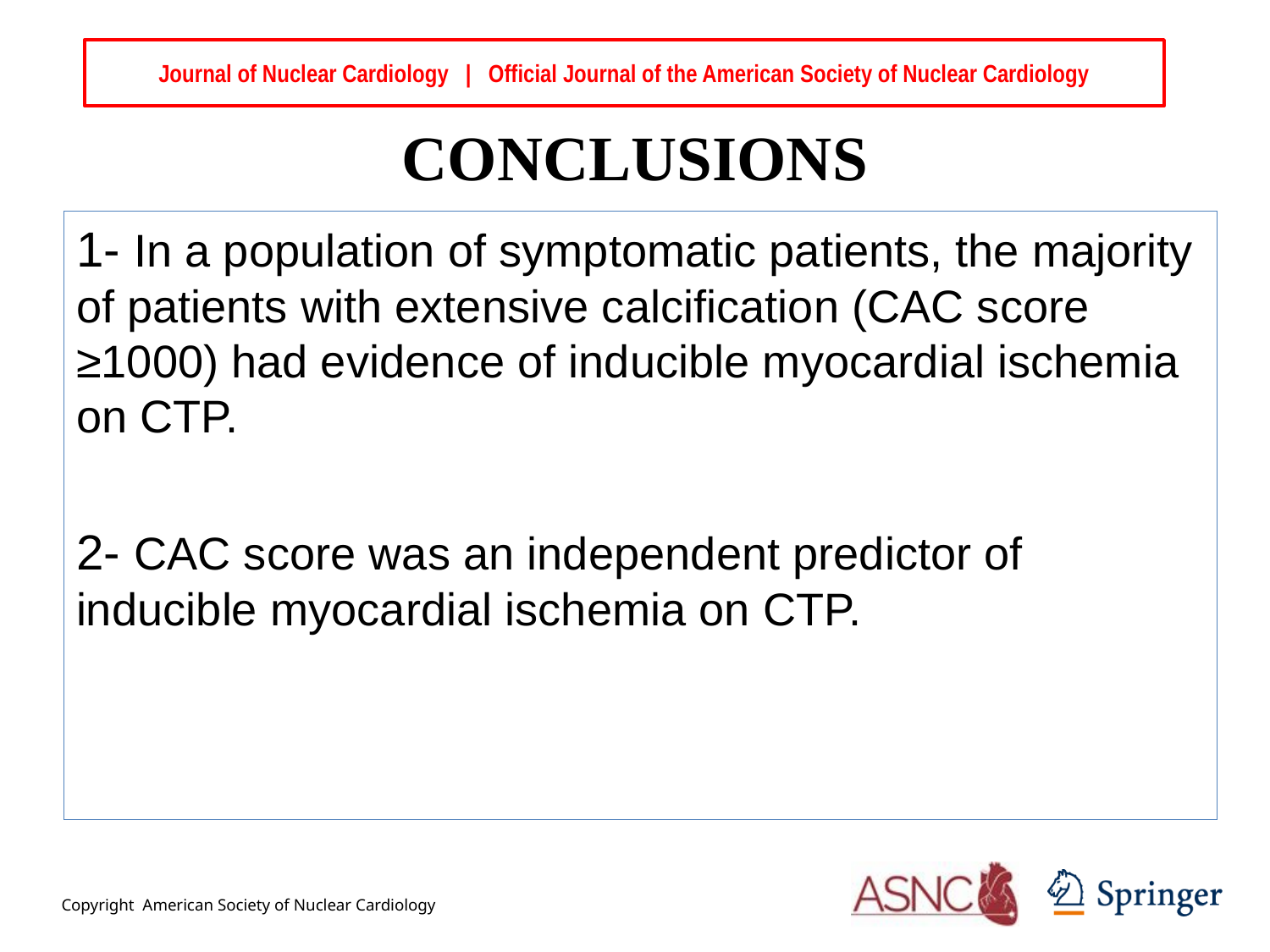

Journal of Nuclear Cardiology | Official Journal of the American Society of Nuclear Cardiology
# CONCLUSIONS
1- In a population of symptomatic patients, the majority of patients with extensive calcification (CAC score ≥1000) had evidence of inducible myocardial ischemia on CTP.
2- CAC score was an independent predictor of inducible myocardial ischemia on CTP.
Copyright American Society of Nuclear Cardiology
